# Electrochemical sensors, MTT and immunofluorescence assays for monitoring the proliferation effects of *cissus populnea *extracts on Sertoli cells

**DOI:** 10.1186/1477-7827-9-65

**Published:** 2011-05-16

**Authors:** Elizabeth Osibote, Naumih Noah, Omowunmi Sadik, Dennis McGee, Modupe Ogunlesi

**Affiliations:** 1 Department of Chemistry, Center for Advanced Sensors & Environmental Monitoring, State University of New York at Binghamton, NY 13902-6000, USA; 2 Department of Biological Sciences, State University of New York at Binghamton, NY 13902-6000, USA

## Abstract

**Background:**

We describe the development of an electrochemical sensor array for monitoring the proliferation effects of cissus populnea plant extracts on TM4 Sertoli cells.

**Methods:**

The proliferation activities of the extracts on Sertoli cells were studied using a high-throughput electrochemical sensor array (DOX-96) and the analytical sensor characteristics were compared with conventional colorimetric MTT (3-(4,5-dimethylthiazol-2-yl)-2,5-diphenyltetrazolium bromide) assay and fluorescence spectroscopy.

**Results:**

This work shows that there is a definite positive trend in the proliferation effect of the extract of *Cissus populnea *on the TM4 Sertoli cells. All of the three techniques confirmed that the most effective concentration for the proliferation is 10 ppm. At this concentration, the proliferation effect was established around 120% for both DOX-96 and MTT techniques, whereas fluorescence assays showed a higher level (120-150%). DOX-96 showed a lower limit of detection (1.25 × 10(4) cells/ml); whereas the LOD recorded for both MTT and fluorescence techniques was 2.5 × 10(4) cells/ml. Visual examination of the cells by means of confocal fluorescence microscopy confirmed the proliferation of Sertoli cells as was determined using the MTT assay. This investigation provides a confident interpretation of the results and proved that the most effective concentration for the proliferation using *Cissus populnea *plant extract is 10 ppm.

**Conclusions:**

Overall, the DOX results compared well with the conventional methods of checking proliferation of cells. The fascinating feature of the sensor array is the ability to provide continuous proliferation experiments with no additional reagents including 96 simultaneous electrochemical experiments. The use of the DOX-96 could reduce a typical bioassay time by 20-fold. Thus the DOX-96 can be used as both a research tool and for practical cell culture monitoring.

## Background

*Cissus populnea *is one of the several climbing tropical shrubs that are believed to promote fertility in males and females, although the mechanism is unclear [1]. Another member of the family (*Cissus sicyoides*) has been reported for the treatment of rheumatism, epilepsy and stroke [2]. Extracts from the plant have been screened for antimicrobial activities [3], treatment of trypanosomiasis [4], as hepatoprotective agent [5] at low dosage and as hepatotoxic agent at high dosage [5,6]. The extracts are also believed to exhibit hypoglycemic and antilipemic effects [2] as well as remedy for anti-sickling properties [7].

Extracts from the stem of *Cissus populnea *are believed to improve fertility in men with low sperm count [1]. Although widely used in the West African region as profertility plant, the in vitro activities on sex cells have not been reported [1,5]. Testicular functions are known to be primarily regulated by luteinizing hormone (LH) and follicle stimulating hormones (FSH) [8]. FSH supports the growth of Sertoli cells and abnormal FSH levels in both male and female can be related to infertility. Sertoli cell line from the reproductive organs of the male rat was used for this study because of its unique ability to communicate with all germ cell generations and with the myoid cells in the reproductive system [9]. The cells respond to FSH to produce the testosterone needed for reproduction and their products assist germ cells through the three phases of spermatogenesis [10]. It is believed that the hormonal regulation of spermatogenesis is mediated by the Sertoli cells since they either partially or completely surround every germ cell [11].

Conventional techniques for monitoring cell proliferation include spectrophotometric methods, fluorescent microscopy, flow cytometry and specialized fluorescence instruments such as plate readers. Each method has its own advantages and disadvantages. For example, the microscopy technique requires extensive sample preparation. These techniques provide indirect approach to monitoring proliferation and cytotoxicity so the measurement errors are considerably increased. Flow cytometry provides a means for scanning one cell at a time and up to 1000 cells per second [12,13]. Information from flow cytometry can be further enhanced by using "cocktails" of dyes at different wavelengths [12,13] but the samples must be measured one at a time, thus increasing the measurement time [14]. Potentiometric probes, such as rhodamines and anionic oxonols, exhibit potential-dependent changes in their trans-membrane distribution that are accompanied by a fluorescence change and are capable of discriminating between live and dead cells. However, there is no correlation between the changes in fluorescence and the exact number of live and dead cells when results are compared to plate counts [15]. Thus conventional approaches are time-consuming, in some cases, requiring the need for additional chemical reagent, which may create interfering background signals. More importantly, there is no current method suitable for continuous monitoring or cell viability, proliferation or cytotoxicity. Thus there is a need to develop new techniques that can assist basic researchers in studying the interactions of natural or exogenous reagents with cells.

We hereby report the development of an electrochemical microelectrode array equipped with 96-microtitre well or DOX-96 for monitoring the proliferation activity of the plant extracts. DOX-96 is a multi-channel electrochemical sensor designed to measure dissolved oxygen content in 96-well plates [16,17]. The system has been successfully tested for determining the minimum inhibition concentrations (MIC's) for various antibiotics/cell combinations showing a 98.2% agreement with the standard microdilution method [18]. DOX-96 multielectrode array system provides a low-cost, rapid, and high throughput method of accessing the proliferation effects of the Sertoli sex cells. The system is a portable electrochemical measurement that is equipped with a multipotentiostat and it allows 96-individual electrochemical experiments with three electrodes embedded in each well. Unlike colorimetric or fluorimetric measurements, it operates without the use of an added chemical reagents since it is based on the consumption (or lack thereof) of oxygen by the cells. For cells growing in medium, the DOX-96 provides a sharp, steady decrease in dissolved oxygen as the cells grow. To our knowledge, this is the first report studying proliferation effects of the extracts from the plant *Cissus populnea *on Sertoli cells. This study may have implications in the profertility actions of the plant extracts as well as the use of low-cost sensors for biological monitoring, especially in regions with limited research resources.

## Methods

### Materials

Methanol, ethylacetate, butanol, hexane, penicillin/streptomycin and 2 Mm L-glutamine were purchased from Fisher Scientific (Pittsburgh, PA). (3-(4,5-dimethylthiazol-2-yl)-2,5-diphenyltetrazolium bromide(MTT) with solutions B and C were obtained from Millipore Corporation (Billerica, MA). SYTO 24 green was purchased from Invitrogen Molecular Probes. The Sertoli cell line TM4 (ATCC CRL 1715 with Lot # 4328836) was obtained from American Type Culture Collection (Manassas, VA). Tissue culture medium (TCM) containing Ham's F12 nutrient was purchased from Sigma-Aldrich, (St. Louis, MO). 10% Fetal Bovine Serum (FBS) was from HyClone Laboratories, Inc. (South Logan, UT). All measurements were carried out using Nanopure water with resistivity of 18 MΩ or better.

### Stock solutions

Dubellco's phosphate buffered saline (DPBS) (lot #092K83011) powder was dissolved in water at 15-20°C with continuous stirring. The solution was then adjusted to pH 0.3 and filtered using 0.22 μm cellulose acetate (lot#28408504).

***MTT ***stock solution was prepared by dissolving 50 mg of the crystals in 10 ml of DPBS and filtered before use. For the Syto 24 green, the reagent was diluted with DPBS 10 times before being used for the fluorescence assay. 10 μl of Syto 24 green was mixed with 90 μl of DPBS to make 100 μl solution.

***Tissue culture medium (TCM) ***was prepared with Ham's F12+DMEM 1:1 +2 mmole glutamine (lot # ATA 31140), 5% horse/equine serum (25 ml for 500 ml of DMEM), 2.5% fetal bovine serum (12.5 ml for 500 ml DMEM) and 0.5% penicillin/streptomycin ( 2.5-5 ml for 500 ml DMEM). This mixture was filtered with sterile filter and the pH adjusted to pH 7.4 using 1 M sodium hydroxide.

### Preparation of extracts

Fresh stems of *cissus populnea *plants were obtained from local market in Lagos. They were identified and authenticated at the forestry research institute of Nigeria (FRIN) and subsequently assigned the voucher specimen number FHI 108222. The stems were cut and dried at room temperature and grounded to powder which was subsequently soaked in 50% methanol/water with continuous stirring for 72 hrs to obtain 50% methanolic extract. Extract was removed, rinsed and filtered under vacuum before evaporating to dryness at 40°C using a rotary evaporator. The dried methanol extract was dissolved in water and partitioned into three fractions (hexane, ethylacetate and butanol) which were evaporated to dryness with a vacuum oven at 40°C. Various concentrations ranging from 1-100 ppm were obtained from the fractions. Isolation, extraction and structural characterization of the plant extracts were carried out using UV/Visible spectroscopy (Additional file 1, Table S1) flash chromatography, HPLC, GC/MS (Additional file 2, Figure S1, Additional file 3, Figure S2, Additional file 4, Table S2) and ^1^H NMR.

### Cell culture

The Sertoli cell line -TM4 was cultured as described in literature [19]. The tissue culture medium (TCM) was contained DMEM + Ham's F12 (1:1) nutrient with 1.5 g/L sodium bicarbonate supplemented with 5% horse serum (HS); 2.5% fetal bovine serum (FBS); 100 U/ml penicillin/streptomycin and 2 mM L-glutamine. The cell culture was incubated at 37°C with 5% CO_2 _in air atmosphere. Cultures grown in a conventional 96 well plate were used for both MTT and fluorescence assays. Before plating, the number of cells was determined using the trypan blue staining assay. The cell concentration used for the assay was 2.5 × 10(4) cells per well after optimization.

## Instrumentation

The incubator (Iowa Model #460) used was supplied by Barnstead Lifeline International, Dubuque. Absorbance and fluorescence measurements were carried out using a multi-well plate fluorimeter CytoFluor II Fluorescence reader set at an excitation wavelength of 485 nm and an emission of 528 nm. A dual ultra micro-plate reader, model ELX808, BioTEK Instruments Inc., was used to measure the absorbance (*A*570-650 nm) for proliferation measurements using the MTT protocol while the fluorescence was measured at 485 nm. The confocal microscopy data was obtained using olympus fluorescence microscope at a magnification of 200_x _The DOX-dissolved oxygen multielectrode potentiostat/microplate wells with the electrodes were supplied by Daikin Corp., Japan.

### DOX-96 oxygen sensor

The DOX-96 system uses a conventional 96-well plate with three disposable gold plated electrodes (reference, working, and auxiliary) inserted in each well. Cell viability and proliferation measurements using the DOX-96 system is based on continuous measurement of dissolved oxygen levels with no added reagents. The use of this system for cell proliferation measurements involves a careful optimization of all the operational parameters. These include stability and reproducibility, applied potential, cell density per well, volume of tissue cultured media per well, incubation and total measuring time, temperature, and sterilization procedure.

#### Electrochemical protocol

The protocol for measuring cell proliferation using the DOX biosensor involves growing the cells into optimum culture media containing the selected plant extracts for specific period (24, 48, and 72 hours). Following a desired time of exposure to the extracts, the 96 electrodes were placed onto the top of a 96-well plate. These electrodes were designed to be disposable and easy to fit into a conventional 96-well cell culture plates. Reusing these electrodes for 2 to 3 times were possible if cleaning and autoclaving is directly applied immediately following usage. The 96-well plates including the electrodes were inserted into the DOX instrument, which resembles a small optical microplate reader. Prior to the measurements, the DOX device was placed inside the incubator for 30 min. This step ensured that the cells received the optimum cell culture conditions (5-10% CO_2_, 90% humidity, and a constant temperature of 37°C) during measurements. The applied potential that gave best current results with TM4 cells were then chosen for all cells used in this study. The registered current at the end of measurements time was monitored using the DOX-96 accompanying software. Current reading after 50 minute of continuous monitoring (at 3000 second) was chosen for quantitation and comparison of the DOX results with the other approaches. Results were correlated with the amount of cells, optical density (OD), and also with the level of plant extract of each well.

#### Optimization

The DOX sensors were first optimized in order to obtain the best concentration of the cells to be used. This was carried out by putting the TM4 cells in a 96-well plate at various concentrations. The concentration at which the cells gave the best and stable current was then chosen for measurements which were 1.25 × 10(4) cells/ml. The potential was also optimized by running the experiments at different potentials (-300 mV, -400 mV and -700 mV) and the potential that gave the most stable current with time selected for the actual measurements. After optimizing the concentration of the cells, specified concentration of TM4 cells were placed in a 96 well plate at the optimized concentration per well and 200 μL of TCM was added. The cells were then incubated for 24hrs using the same conditions as the MTT to reach 70% confluence. After 24 hrs, changes in the oxygen level of selected wells were measured using a conventional 96-well plate by placing 200 μl of growth medium with or without cells in the presence or absence of extracts. Then, 96 gold-plated electrodes were fitted onto the top of the plate covered with the cell solution and placed in the measurement chamber of the sensor where the oxygen reduction current was monitored continuously at a fixed potential of -300 mV for 1 h. This corresponds to a maximum value of the oxygen reduction current. Negative control wells loaded with 200 μl of broth only were tested for each experiment. Each experiment was simultaneously repeated four times. The electrodes were used as disposable devices and each plate was used for a single measurement.

### Proliferation measurement

The Sertoli cells were plated at a density of 2.5 × 10(4) cells per well in 100 μl of TCM. For these experiments, three similar plates containing TCM and/or cells were prepared simultaneously following the same experimental procedure. Studies of the effect of the plant extracts on the growth of the TM4 Sertoli cells were carried out by adding 2 μl of plant extract solutions (at the corresponding concentration) to each well containing cells grown for 1 day (at about 70-80% confluence). The plates were incubated for 24, 48 and 72 hours after the extracts were added and cell viability was tested using the two protocols: MTT and fluorescence assays. The control contained the solvents used in the fractionation process but with no plant extracts.

#### MTT assay

The proliferation activities of the plant extracts on TM4 Sertoli cells were first determined using the standard micro-plate colorimetric MTT assay. 5 mg/ml MTT reagent was prepared in phosphate-buffered saline (PBS) to obtain a stock solution; it was filtered and ready for use. 5 μl of the stock solution was added to each well containing the cells in 50 μl of medium, and the plates were incubated for 4 h under regular growth conditions at 37°C with 5% CO_2 _in air atmosphere. 200 μl of acidified 2-propanol (isopropanol with 0.04 N HCl) was added to each well and mixed in order to dissolve the precipitate and to ensure color development. The HCl in the isopropanol converts the phenol red in the TCM to a yellow color that does not interfere with MTT formazan measurement. The absorbance at 570 nm was measured on a dual micro-plate reader with the absorbance at 650 nm subtracted to account for the plastic well and cellular debris. To avoid interferences (such as the presence of solvents used to dissolve the extracts or the intrinsic plant polyphenols) with MTT, the extracts on the Sertoli cells were first washed before adding the MTT reagent. For these experiments, after the supernatant (TCM containing solvents or excess extracts) was removed, the cells were washed twice with fresh tissue culture medium before the addition of 5 μl of MTT. Finally, 50 μl of TCM was added to each well. Results are expressed and calculated as R_n _(i.e. the residual activity of well in which extract was added). Proliferation data are expressed as percentages of the residual response using 100% sample in the absence of extracts but containing the same amount of the solvents.

#### Fluorescence assay

The proliferation effect of the extracts on the cells was also determined by measuring the level of DNA synthesis after staining the cells with a fluorescent dye. For these tests, a similar experimental procedure was used to study the proliferation (i.e. extract concentrations and incubation times) as for the MTT method. The level of DNA was determined by measuring the fluorescence intensity of the cells containing 5 μl per well solution of a cell permeant chromophore SYTO 24 green fluorescent nucleic acid stain (diluted 1/100 in PBS). For these experiments, the final volume of cell solution per each well was 100 μl. After 20 min of incubation with the cells, the fluorescent dye was incorporated into the cells exhibiting bright, green fluorescence following its binding to DNA. The corresponding fluorescence was measured in a fluorescence multi-well micro-plate reader or by fluorescence microscopy. The cells were visualized using an Olympus fluorescence microscope at a magnification of 200_x_. Background fluorescence was considered for wells containing TCM with extracts in the absence of cells. The percentage of residual response was expressed considering a control sample containing untreated cells exposed to the same amount of solvent in which the extracts was added. Results are presented as percentages of residual activity by considering as 100% a control sample containing cells with the same amount of methanol but in the absence of extracts. The fluorescence was measured at 485/528 nm.

## Results and discussion

The overall goal of this work was to demonstrate the enhanced proliferation effects of the Sertoli cells upon exposure to the plant extracts. The immediate objective was to compare the performance of three analytical techniques on the proliferation effects: electrochemical sensor arrays (DOX-96), colorimetric assay using MTT and fluorescence techniques. All three approaches involved different detection mechanisms.

### Spermatogenesis and proliferation effect of cells

Follicle stimulating hormone (FSH) supports the growth of Sertoli cells and abnormal FSH levels in both male and female can be related to infertility. Sertoli cell lines from the reproductive organs of the male rat was used for this study because of its unique ability in the reproductive system to communicate with all germ cell generations and with the myoid cells [9]. Sertoli cells can also produce important growth factors and cytokines that support the development of the male germ cells. It is believed that the hormonal regulations of spermatogenesis is mediated by the Sertoli cells since they either partially or completely surround every germ cell [11].

**Figure 1:**
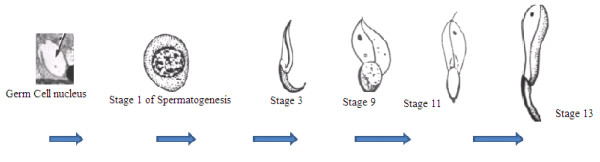
**Stages involved in the spermatogenesis process.**.

There are fourteen main stages in the spermatogenetic process but stages 1 and 2 can be taken as the beginning of the process, 3-7 are not much of a difference and degeneration starts from stage 8, proceeding up to 13 till the matured sperm cells are formed. The arrow shown in Figure 1 indicates the direction of the spermatogenetic process. Stages 1-7 do not contribute much to the matured cells and have been skipped. As degeneration increases, the lipid content increases and the germ cells matures. It is believed that some variation in the structure and function of Sertoli cells is associated with the spermatogenic wave [13]. It has been widely reported that the hormonal regulation of spermatogenesis is mediated by the Sertoli cells since they either partially or completely surround every germ cell [11,20-22].

Although germ cells are under the control of Sertoli cells, they can exert feedback actions on them. Germ cells are continually renewed and the Sertoli cells that cease to divide during the pubertial development in most mammals form the seminiferous epithelium. At any point in the tubule, several germ cells develop simultaneously in contact with Sertoli cells from the base to the apex of the epithelium. These play a major role in the establishment and maintenance of spermatogenesis [10]. Therefore the rationale for selecting the Sertoli cell line for this study is that it provides a direct response to proliferation effect of the extracts from the plant. Additionally, Sertoli cells were shown to respond to FSH and could therefore serve as an ideal platform to demonstrate the proliferation effects of the plant extract [9,23].

### MTT measurements

Treatment of cells with MTT results in a dark blue formazan product generated via the reduction of MTT by mitochondrial dehydrogenases in living cells. The absorbance was taken at 570 nm.

The residual activity is derived from the equation(1)

where *A*_s _is the absorbance value obtained for a sample containing cells in the presence of a given concentration of extract and *A*_0 _is the absorbance value corresponding to a well with cells containing TCM and no extract added. The MTT results represent the means (SD from tests carried out in five wells with cells from at least three independent experiments using cells from different cultures).

**Figure 2:**
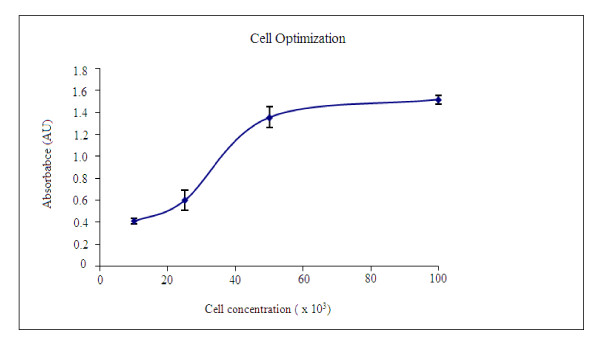
**UV/Vis spectroscopic cell optimization for MTT and fluorescence technique. **Absorbance was measured at 527 nm for MTT and 485 nm for fluorescence. Optimum cell culture conditions: 5-10% CO_2_, 90% humidity and a constant temperature of 37°C.

**Figure 3:**
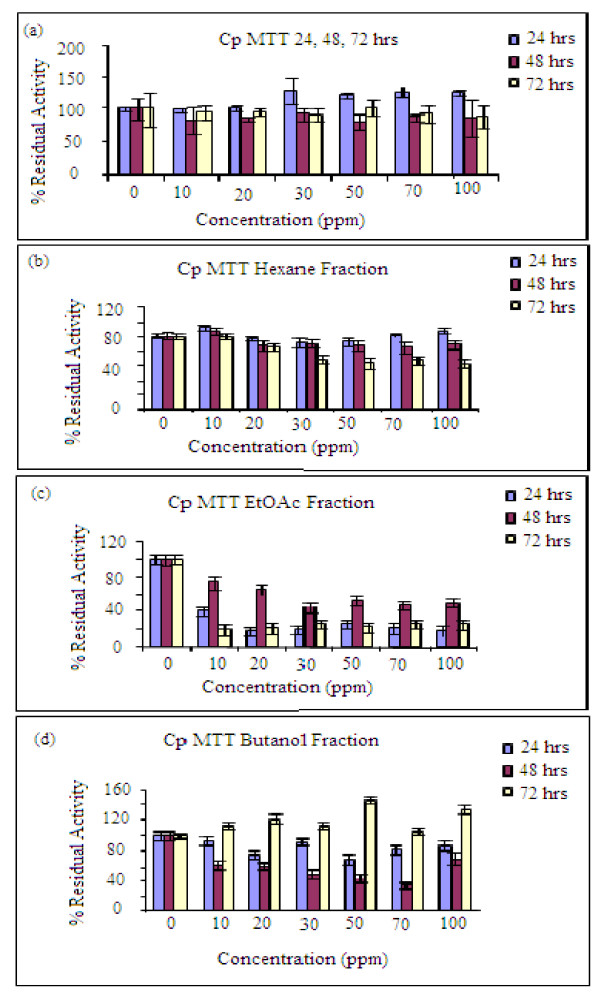
**MTT results showing the residual activity taken at normal incubation conditions for 4 hrs in air atmosphere at concentration of 10 ppm intervals. **(a) Crude extracts (b) hexane fraction (c) ethyl acetate fraction (d) butanol fraction. The absorbance was taken at 570 nm, 200 uL of acidified 2-propanol with 0.04 N HCl was added to each well and mixed in order to dissolve the precipitate and ensure color development.

First the absorbance of different cell concentrations for the MTT assay was taken in order to determine the best cell concentration. The absorbance is required to be less than 1 and therefore as shown in Figure 2, the cell concentration which gave slightly less that 1.0 absorbance unit was 2.5 × 10(4). This serves as the cell concentration contained in each well that was used for the entire MTT and fluorescence assays. The MTT assay results (Figure 3a) showed that the whole extract had proliferation effect only at 24 hrs incubation and the residual activity was above that of the control between 20 and 100 ppm (120-140%) with the highest residual activity at 30 ppm. After 48 and 72 hrs incubation, the residual activities were lower than that of the control. This is attributed to the effectiveness of the proliferation activity reducing as time/concentration increases. The effect of concentration increase should ultimately result in toxicity as reported by Ojiako [24] which may relate to the amount used per body weight.

The extracts were separated into three fractions (hexane, ethylacetate and butanol) and were spiked separately onto the cells in order to determine which fractions will have the most significant effect on cell proliferation. This procedure was also carried out in order to assess any possible synergic effects of the extracts on the cells. MTT results showed that the hexane fraction (Figure 3b), produced residual activity ranging between 105 and 110% at 10, 70 and 100 ppm following 24hrs incubation, while the other concentrations gave residual activities similar to the control. At 48 and 72hrs, the residual activities dropped to 80% and 60% respectively. The ethyl acetate fraction (Figure 3c) produced residual activities that were lower than that of the control at all the concentrations and incubation times tested. The ethyl acetate fractions when tested alone resulted in absorbance and fluorescence values that were higher than that of the control, so the result from the fraction was not used for the proliferation studies.

For the butanol fraction (Figure 3d), MTT result showed the most proliferation effect at 72 hrs and the effect rose gradually to the maximum at 50 ppm. The residual activity rose from 110-140% of the control. At lower incubation times (24, 48 hrs) there was no proliferation as the residual activities were lower than those of the control (<100%). The residual activities for the butanol fraction at 72h of incubation were however not consistent so the 24h results were used for subsequent analysis.

**Figure 4:**
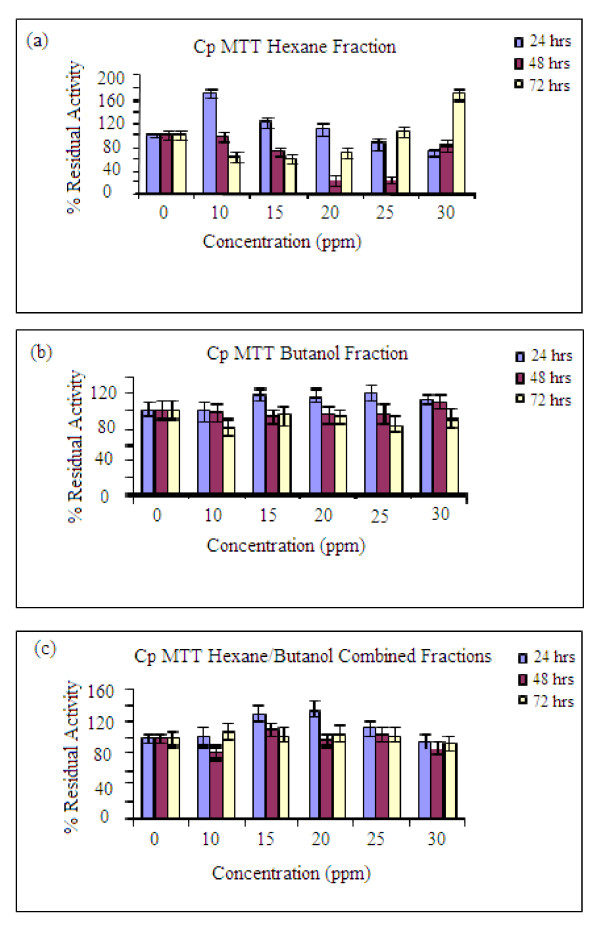
**MTT results for the residual activities of the fractions that are active (a)-Hexane fraction, (b) Butanol fraction, (c) Combined hexane fractions. **Other conditions as in Figure 3.

At lower concentrations (10, 15, 20, 25 and 30 ppm) of the spiked extracts, the proliferation effect became more noticeable. The residual activities obtained for hexane fraction were 110-120% at 24 hrs while for the butanol fraction all the concentrations gave values between 105-110%. This observation might support the hypothesis that the plant extract contains active ingredients that will induce enhanced proliferation in the short-term, which perhaps will decrease or even result in toxicity upon prolonged exposure. Combining the hexane and butanol fractions, the effect was found to be more noticeable around 10 ppm concentrations with residual activity between 105-120% over that of control (Figure 4a-c). The proliferation effect was found to gradually increase from 1 ppm to 10 ppm and dropped drastically at 15 ppm for the hexane fraction; similar effect was recorded for the butanol fraction (Figure 4a-b). It should be noted that the extracts are most likely to give the proliferation effects at stages 8-14 during which degeneration of the cells are more noticeable and spermatogenesis progresses (Figure 1). The pattern of germ cell degeneration in the normal rat seminiferous epithelium is significantly altered when the availability of testosterone is reduced or completely withdrawn when germ cells at stage 7 are the first to degenerate and do so in large numbers compared with all other stages. These findings suggest that stage 7 in particular acutely depends upon an adequate supply of testosterone [11]

### Fluorescence assays

The fluorescence result was calculated as percentage of residual activity using the following equation:

where *F*_s _is the fluorescence determined for a sample containing cells in the presence of the extracts, *F*_s_^b ^is the background fluorescence determined in wells with the same amount of extracts but in the absence of cells, *F*_o _is the fluorescence value corresponding to a well with cells in TCM with the same amount of solvent in which the extract was added, and *F*_o_^b ^is the background fluorescence in wells with TCM and solvent only.

**Figure 5:**
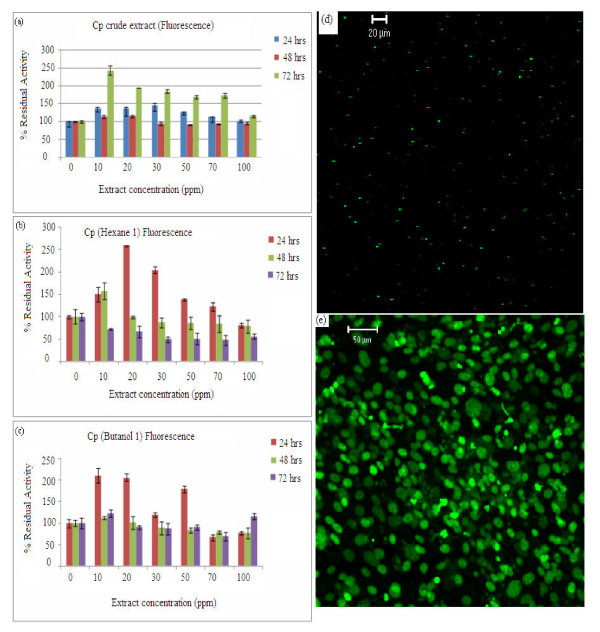
**Fluorescence assay results for the residual activities of the crude extract and the fractions at concentrations of 10 ppm intervals. **(a) Crude extract, (b) Hexane fraction, (c) Butanol fraction, (d) Visual morphology of the TM4 cells using confocal microscopy without plant extracts, (e) Visual morphology of the TM4 cells using confocal microscopy with plant extracts. The cells were incubated at regular growth conditions at 37°C and 5-10% CO_2 _in air atmosphere. Excitation wavelength was 485 nm and emission wavelength was 528 nm. Other conditions are as Figure 3.

Results are presented as the average SD from at least three independent experiments in five different wells. The fluorescence results (Figure 5a) shows the proliferation effect for the whole extract in methanol/water. It was observed that the residual activity was about 150% at 24 hrs incubation. The fluorescence assay definitely showed enhanced effect for the extracts with about the same trend over the control for both the crude extract and the fractions. The trends are similar to that of the MTT but at higher magnitude. The variation observed is attributed to the fact that MTT assay is more of a direct measurement because it measures the cells activity directly while the Fluorescence assay tends to show background effects. The hexane fraction (Figure 5b) had the proliferation effect at all the concentrations with the residual activity above 120% at 24 hrs incubation.

For these extracts, some proliferation effects were noticed at 10 ppm at 48hrs incubation but for the others the percentage proliferations were much lower than the control. The butanol fraction (Figure 5c) produced noticeable effect above the control except at 70 ppm at 24hrs incubation, correlating to stages 7-13 as shown in Figure 1. This effect could be attributed to the presence and/or absence of testosterone as reported by Kerr et al [20,21]. The cells were visualized using an Olympus fluorescence microscope at a magnification of 200_x_. Figure 5(d/e) shows the confocal microscopic pictures of the Sertoli cells before and after exposure to the plant extracts.

**Figure 6:**
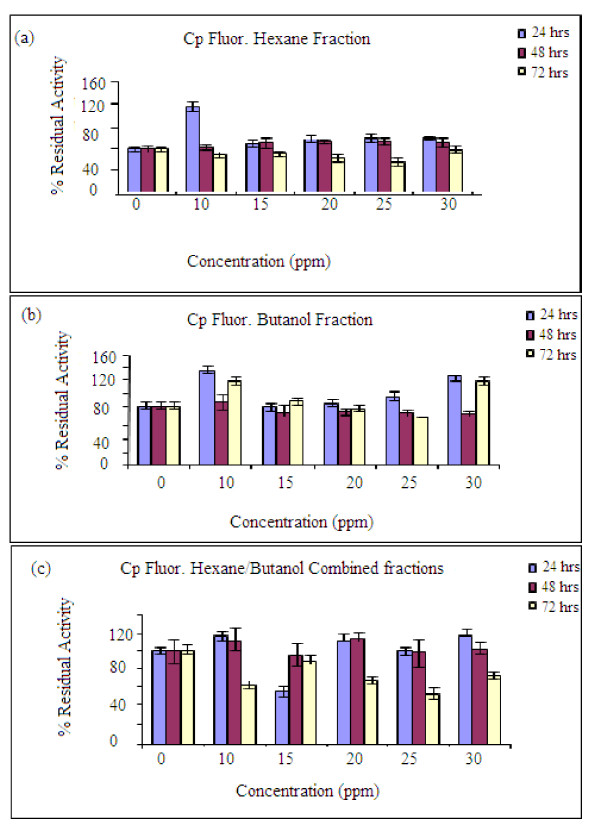
**Fluorescence assay results for the residual activities of the active fractions at smaller concentration intervals. **(a) Hexane fraction, (b) Butanol fraction, (c) combined/mixed fractions of hexane and butanol. Optimum cell culture conditions: 5-10% CO_2_, 90% humidity and a constant temperature of 37°C. Other conditions are as in Figure 5.

At lower concentrations: 2 - 15 ppm (Figure 6a), the hexane fraction showed proliferation effect at 24 hrs incubation with values ranging between 120 and 180% of the control, and no effect was recorded 48 and 72 hrs respectively. The butanol fraction (Figure 6b) also showed more effect at 24 hrs than 48/72 hrs with values raging from 105-150%. Combining the two fractions (Figure 6c) the proliferation effect was also higher at 24 hrs incubation with values between 110-120%. 10 - 20 ppm produced the highest values for at 24 and 48 hrs incubation. However, a reduction effect was noticed as the concentration increased after 72 hrs and no proliferation effect was recorded [24]. Overall, the most significant proliferation effect was recorded at 10 ppm spiked extract concentration. Based on these observations, subsequent experiments were carried out at lower concentrations in order to observe possible trends at smaller increases (15-30 ppm) in extract concentrations.

The MTT and florescence results showed that the highest proliferation effects for both the hexane and the ethanol fractions was ~10 ppm at 24 hrs and this trend reduced as the concentration increased. This result confirmed that the highest proliferation level was ~10 ppm. In addition, it is worth noting that for the hexane extract, the highest residual activity was 7 ppm (MTT). A gradual increase in proliferation activity was noticed with very little change in concentration reaching a sharp peak at ~7 ppm, followed by a noticeable decline. Overall, it was observed that the EC_50 _(i.e. the molar concentration of an agonist, in this case the plant extract; which produces 50% of the maximum possible response for that agonist) for the hexane extract was 5.8 ppm. As previously reported by Neubig et al [25], a small increase in concentration will give gradual increase in effectiveness until reaching a maximum effective concentration after which for an agonist, as the concentration further increases, there is a sharp decline in effectiveness.

This work shows that there is a definite positive trend in the proliferation effect of the extract of *cissus populnea *on the Sertoli cells TM4 and at low concentrations (2-15 ppm), the effect is more noticeable whereas at higher concentration (20 - 100 ppm) the proliferation effect is significantly reduced. As noted earlier, there is a long term toxic effect of the *cissus populnea extract *on some tested animals (normal rabbits-Sprague dawley) [1,5]. This correlates well with the current in-vitro studies. The spermatogenetic process as explained in Figure 1 can be used to support the proliferation effect of the extracts on the Sertoli cells. The effect of the extracts will be felt more at stages 8-13 with stage 7 being the critical point at which there is no degeneration of the cells. Further confirmation of the MTT and Fluorescence results was obtained by microscopic examination of the cells exposed upon binding to the fluorescent dye. This approach is also critical to confirming the cell proliferation process. Visual examination of the cells by means of fluorescence confocal microscopy (Figure 5) confirmed the proliferation of Sertoli cells in the wells as was determined using the MTT assay. This investigation provides a confident interpretation of the results and proved that the most effective concentration for the proliferation using Cissus populnea plant extract is 10 ppm.

### DOX measurements

Oxygen is one of the key metabolites in aerobic systems and a good indicator of metabolic activity of cells. Thus, the level of oxygen consumed by the cells can provide information on cell viability. The DOX-96 is a multi-channel electrochemical sensor designed to measure the dissolved oxygen content in 96-well plates [16,26,27]. DOX is fully automated, portable, equipped with a multipotentiostat and can be connected to a computer. The latter enables external control of the instrument, on line recording of experimental parameters, graphical presentation and data storage. The instrument software plots current intensity versus time for each well while data are simultaneously processed for 12 channels, each corresponding to 8 sensors.

**Figure 7:**
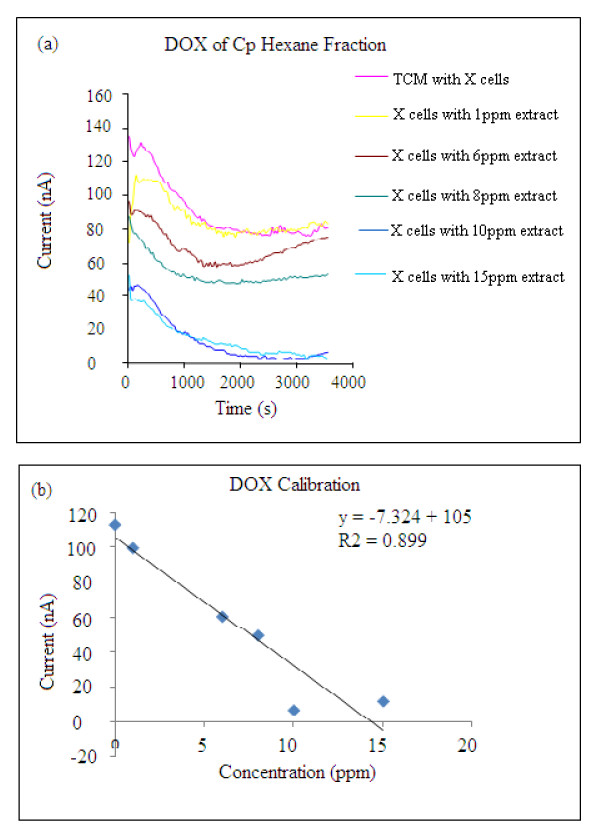
**DOX sensor results. **(a) Hexane fraction containing TM4 cells at varying concentrations of plant extracts. X = 1.25 × 10(4) cell/ml, (b) the calibration curve recorded at 1800 seconds for the DOX sensor array. Optimum applied potential used was -300 mV.

Experimental set-up involves the use of 96 disposable electrodes in a three-electrode format (reference, working and auxiliary) embedded in each well. DOX experiments were initially carried out at different potentials and varying concentrations of the extract in order to determine the optimum experimental conditions. DOX provides information on respiratory activity of cells by determining the current arising from the reduction of oxygen at negative potentials. During cell respiration, oxygen is consumed, and the measured current reduces to a threshold value. The time it takes to achieve this threshold current is proportional to the initial cell concentration. Increasing concentrations of the plant extracts produced proliferation of the cells hence an increase in the cell concentration. At increasing concentration of cells, the cells consume more oxygen and hence the dissolved oxygen decreased which is indicated by a decrease in the current measured at the working electrode (Figure 7). Approximately 20% loss of current signal is noted in the presence of TCM alone. This is probably due to the time needed to achieve equilibration at the sensing electrodes. For this reason the current used to assess the proliferation effect was considered at a constant time, i.e. after 1000 seconds. Thus, any variation taking place in TCM also occurs in the solution containing the cells in TCM

### Comparative analysis of DOX-sensors

DOX result showed a similar trend as for the MTT and fluorescence assays. Comparative analysis of the three monitoring techniques is provided in Table 1. All three techniques confirmed that the most effective concentration for the proliferation is 10 ppm. At this concentration, the proliferation effect was established around 120% for both the DOX and MTT techniques, whereas fluorescence assays showed a higher level. This deviation is attributed to higher background signals resulting from the fluorescent reagent. DOX showed a lower limit of detection (1.25 × 10(4) cells/ml), whereas the LOD recorded for both MTT and fluorescence techniques was 2.5 × 10(4) cells/ml. Thus the electrochemical sensor array provides a better sensitivity compared to the conventional techniques. Overall, the DOX results compared well with the conventional methods of checking proliferation of cells.

**Table 1:** Comparative analytical characteristics for electrochemical, MTT and fluorescence techniques

Technique	Time	Reagents/Measurement	Effective extract conc. (ppm)	Proliferation effect	LOD(cells/ml
DOX	40 min.	• No additional reagents • No background signals • No washing needed for the cells	10	120%	1.25 × 10(4)
MTT	4Hrs	• MTT, Acidified Propanol • Washing step required for the cells	10	105-120%	2.5 × 10(4)
Fluorescence	20min	• Syto 24 Green • There is background effect of the solvents and medium	10	120-150%	2.5 × 10(4)

Glossary: LOD- Limit of detection; MTT-(3-(4,5-dimethylthiazol-2-yl)-2,5 diphenyltetrazolium bromide, DOX- Dissolved Oxygen Multi electrode potentiostat/microplate; Experimental conditions- Cells incubated under regular growth conditions at 37°C with 5% CO_2 _in air atmosphere

In addition, the result of the DOX was faster with no background effect as a result of any added reagent and there is no washing needed for the cells. At a very short time the cells could be monitored as the current varied with time. The fascinating feature of the DOX-96 is the ability to perform 12 simultaneous electrochemical experiments. Each experiment can be duplicated 8 times. This implies that a total of 96 experiments could be carried out at once. The use of the DOX-96 could reduce a typical bioassay time by 20-fold. Thus the Dox-96 can be used as both a research tool and for practical cell culture monitoring; offering significant advantages with respect to assay management in high-throughput systems.

## Conclusions

A suite of analytical techniques has been used to examine the proliferation effects of *cissus populnea plant *extracts on Sertoli cells. Each technique utilizes a different mechanism to assess cell proliferation, with MTT monitoring the mitochondrial enzyme activity in living cells, fluorescence assays focusing on the level of DNA synthesis and DOX based on the metabolic activities of the cells, without the need for added chemical reagents. Further confirmation of the MTT and fluorescence results was obtained by microscopic examination of the cells exposed upon binding to fluorescent dye. This approach is also very important to confirm the cell proliferation process. The fluorescence microscopy confirmed the enhanced number of cells in the wells. In conclusion, the visual examination of the cells by means of fluorescence microscopy confirmed the proliferation of Sertoli cells in the wells as was determined using the MTT and DOX assays indicating the fact that the cell extracts are indeed responsible for the increased proliferation of the Sertoli cells. This investigation provides a confident interpretation of the results and proved that the most effective concentration for the proliferation using Cissus populnea plant extract is 10 ppm. To our knowledge, this is the first study reporting the in-vitro proliferation of Sertoli cells upon exposure to the plant extract. The results obtained from the conventional assays MTT and Fluorescence provided the means of validating the electrochemical assays. All three techniques showed that hexane and butanol extracts from *cissus populnea *have shown proliferation effects on the cells TM4 at low concentration, specifically at 10 ppm.

## Competing interests

The authors declare that they have no competing interests.

## Authors' contributions

EO carried out the plants extraction, characterization using GC/MS, UV/Vis spectroscopy and NMR. NN worked with EO on cell culture and DOX experiments and repeated the cell culture experiments. OAS conceived the sensor concept, designed the project and coordinated the manuscript. DM helped with the confocal microscopy data. MO provided the native plants studied through the support of the Forestry Research Institute of Nigeria (FRIN). All authors read and approved the final manuscript.

## Supplementary Material

Additional file 1 **Table S1**: A table of UV-Visible spectroscopic data Click here for file

Additional file 2 **Figure S1**: Chromatogram of ethylacetate fraction of *Cissus populnea *after derivatization Click here for file

Additional file 3 **Figure S2**: Chromatogram of butanol fraction of *Cissus populnea *after derivatization Click here for file

Additional file 4 **Table S2**: A table of compounds obtained from GCMS analyses of fractions from *Cissus populnea *after derivatization Click here for file
