# Supplementary material for: Electrochemical sensors, MTT and immunofluorescence assays for monitoring the proliferation effects of cissus populnea extracts on Sertoli cells
Source: Reprod Biol Endocrinol. 2011 May 16;9:65. doi: 10.1186/1477-7827-9-65 (PMC3117771; doi:10.1186/1477-7827-9-65)
Supplement: Additional file 1 — Table S1: A table of UV-Visible spectroscopic data [file 1477-7827-9-65-S1.DOC]

**Additional file 1, Supplemental Table S1**

**Table S1:** A table of UV-Visible spectroscopic data

| Absorbance wavelength  (nm) | Functional group likely  represented | *Cissus populnea*  (crude extract) | Hexane  fraction | Ethyl  acetate  fraction | Butanol  fraction |
| --- | --- | --- | --- | --- | --- |
| 175-200 | Alcohols |  |  |  |  |
| 197 198 | Amines |  |  |  |  |
| 200-220 | Amides |  |  |  |  |
| 205 | Esters, Carboxylicacids |  |  |  |  |
| 210-250 | Unsaturated aldehydes |  |  |  |  |
| 210-215 | Sulphides/Thiols |  |  |  |  |
| 245-290 | Aldehydes |  |  |  |  |
| 307 | Ketones | _ |  |  |  |
| 253,263,273 | Trienes | _ |  |  |  |
| 270-290 | Benzoic acids (carbonyls) |  |  |  |  |
| 340-390 | Azo groups |  |  |  |  |
| 215, 305 up to 750 | Phenolics, Flavonoids |  |  |  |  |

UV-VIS data identification from methanol extract of Cissus populnea
