# Supplementary material for: Electrochemical sensors, MTT and immunofluorescence assays for monitoring the proliferation effects of cissus populnea extracts on Sertoli cells
Source: Reprod Biol Endocrinol. 2011 May 16;9:65. doi: 10.1186/1477-7827-9-65 (PMC3117771; doi:10.1186/1477-7827-9-65)

**Additional file 2, Supplemental Figure S1**

**Figure S1:** Chromatogram of ethylacetate fraction of *Cissus populnea* after derivatization


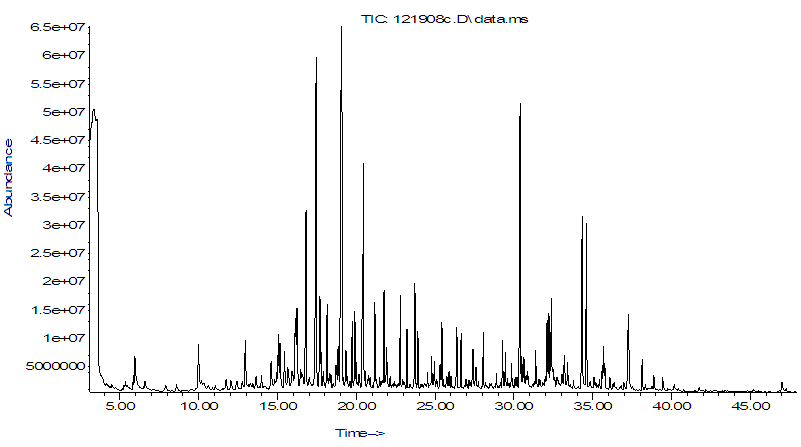

Supplement: Additional file 2 — Figure S1: Chromatogram of ethylacetate fraction of Cissus populnea after derivatization [file 1477-7827-9-65-S2.DOC]
