# Supplementary material for: Electrochemical sensors, MTT and immunofluorescence assays for monitoring the proliferation effects of cissus populnea extracts on Sertoli cells
Source: Reprod Biol Endocrinol. 2011 May 16;9:65. doi: 10.1186/1477-7827-9-65 (PMC3117771; doi:10.1186/1477-7827-9-65)
Supplement: Additional file 3 — Figure S2: Chromatogram of butanol fraction of Cissus populnea after derivatization [file 1477-7827-9-65-S3.DOC]

**Additional file 3, Supplemental Figure S2**

**Figure S2:** Chromatogram of butanol fraction of *Cissus populnea* after derivatization

**
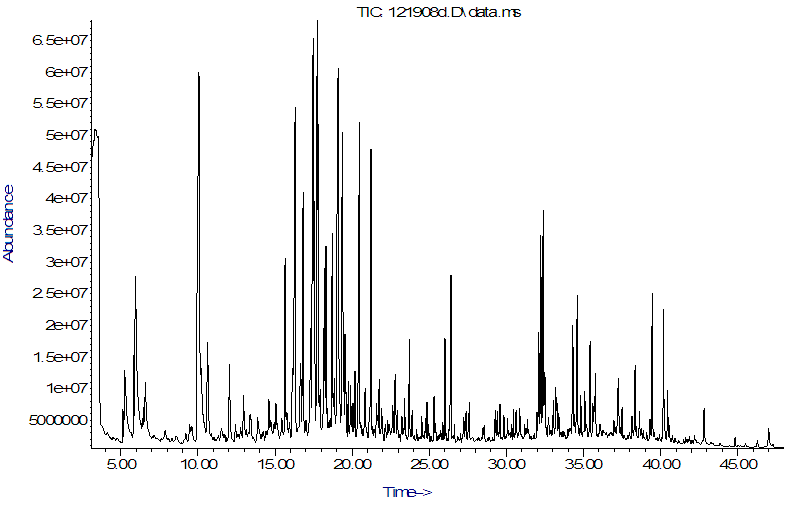
**
