# Supplementary material for: Electrochemical sensors, MTT and immunofluorescence assays for monitoring the proliferation effects of cissus populnea extracts on Sertoli cells
Source: Reprod Biol Endocrinol. 2011 May 16;9:65. doi: 10.1186/1477-7827-9-65 (PMC3117771; doi:10.1186/1477-7827-9-65)
Supplement: Additional file 4 — Table S2: A table of compounds obtained from GCMS analyses of fractions from Cissus populnea after derivatization [file 1477-7827-9-65-S4.DOC]

Additional file 4, Supplemental Table S2

Table S2: A table of compounds obtained from GCMS analyses of fractions from *Cissus populnea* after derivatization

| S/N | R_t_ (min) | Name of compound | Hexane fraction | Ethylacetate fraction Abundance (%) | Butanol fraction Abundance (%) | Remarks |
| --- | --- | --- | --- | --- | --- | --- |
| 1 | 5.25 | Glycerol |  | 0.10 | 0.169 | Used in pharmaceutical formulations, anti-freeze properties sweet but doesn’t raise blood sugar |
| 2 | 5.85 | Butanedioic acid |  | 0.114 | 0.446 | Dicarboxylic acids known to be antibacterial agents |
| 3 | 6.42 | 2,3-bis(hydroxyl)-Propanoic acid |  |  | 0.195 |  |
| 4 | 6.59 | 2-Butenedioic acid(E)- |  | 0.128 | 0.223 | Dicarboxylic acids known to be antibacterial agents |
| 5 | 8.59 | 1-hydroxyl-2- methoxy-Benzene |  | 0.165 |  |  |
| 6 | 9.99 | Butanedioic acid,2,ester |  | 0.803 |  | Dicarboxylic acids known to be antibacterial agents |
| 7 | 10.24 | Malic acid |  |  | 1.064 |  |
| 8 | 10.62 | 1,2,3,4-tetra(hydroxyl) Butane |  |  | 1.242 |  |
| 9 | 12.04 | Ethylmethyl-malonic acid |  | 0.300 |  |  |
| 10 | 12.44 | Heptanedioic acid |  |  | 0.181 | Dicarboxylic acids known to be antibacterial agents |
| 11 | 12.77 | 3-hydroxyl -Benzoic acid |  | 0.209 |  | Benzoic acid and its derivatives are antibacterial agents |
| 12 | 12.96 | 4-(hydroxyl)-Benzoic acid |  | 0.766 | 0.509 | Benzoic acid and its derivatives are antibacterial agents |
| 13 | 13.16 | β DL-Lyxopyranose |  |  | 0.103 |  |
| 14 | 13.39 | 1,4-lactone-2,3,4,5-Tetrahydroxypentanoic acid |  |  | 0.416 |  |
| 15 | 14.60 | Octanedioic acid |  | 0.580 | 0.370 | Dicarboxylic acids known to be antibacterial agents |
| 16 | 15.06 | 3-Hydroxyl hendecanoate |  | 0.685 |  |  |
| 17 | 15.16 | 3,4-bis(hydroxy)-Benzoic acid-methyl ester |  | 0.730 |  | Benzoic acid and its derivatives are antibacterial agents |
| 18 | 15.65 | Xylitol |  |  | 1.240 | Sugar alcohol can be used to replace sugar but do not raise the blood sugar |
| 19 | 15.77 | 4,Methyl α-D-Glucofuranose |  |  | 0.327 | Sugar |
| 20 | 15.94 | 1-Propane-1,2,3-tricarboxylic acid (Z) |  |  | 0.206 |  |
| 21 | 16.16 | 3-methoxy-4-(hydroxyl)-Benzoic acid |  | 1.390 |  | Benzoic acid and its derivatives are antibacterial agents |
| 22 | 16.31 | N-acetyl-N-L-Glutamic acid |  |  | 3.293 |  |
| 23 | 16.81 | Azelaic acid |  | 2.251 | 1.951 | Dicarboxylic acids known to be antibacterial agents |
| 24 | 17.49 | 3,4-bis(hydroxyl Benzoic acid |  |  | 4.104 | Benzoic acid and its derivatives are antibacterial agents |
| 25 | 17.55 | D-Fructose |  |  | 1.631 | Good energy source for sperm cells |
| 26 | 17.69 | 2-hydroxy-1,2,3-Propanetricarboxylic acid |  | 1.048 |  |  |
| 27 | 17.81 | α-methyl-Glucose- |  |  | 1.900 | Sugar |
| 28 | 18.21 | β-DL-Lyxopyranose |  |  | 1.547 | Sugar |
| 29 | 18.79 | β-D-Galactofuranose |  | 0.206 | 0.207 | Sugar |
| 30 | 18.71 | [(3β,5α,20R)-pregnone-3,17,20,21-tetroyl)tetra(hydroxyl) |  | 0.468 |  | Hormone-like maybe precursor to female hormone, estrogen,can be used to improve female fertility |
| 31 | 19.10 | α-D- methyl-Glucopyranose- |  |  | 4.496 | Sugar |
| 32 | 19.34 | D-Mannopyranose |  | 0.536 |  | Sugar |
| 33 | 19.38 | D-Altrose |  |  | 2.852 | Sugar |
| 34 | 19.56 | α-D-Xylopyranose |  |  | 0.804 | Sugar |
| 35 | 19.74 | p-hydroxy-Cinnamic acid |  | 0.668 |  | Used in flavors and in some pharmaceuticals |
| 36 | 19.76 | m-hydroxy-Cinnamic acid |  |  | 0.373 | Used in flavors and in some pharmaceuticals |
| 37 | 20.19 | Glucitol |  |  | 0.418 | Sugar alcohol can be used to replace sugar but do not raise the blood sugar |
| 38 | 21.11 | O,O,O-Pantothenic acid |  |  | 0.152 |  |
| 39 | 21.17 | 3,4-bis(hydroxy)-Cinnamic acid-methyl ester |  | 1.004 |  | Used in flavors and in some pharmaceuticals |
| 40 | 21.23 | Talose |  |  | 1.861 | Sugar |
| 41 | 21.59 | D-Fructose |  |  | 0.458 | Good energy source for sperm cells |
| 42 | 21.76 | Hexadecanoic acid |  | 0.950 | 0.369 | Antifungal properties (in all long chain fatty acids) |
| 43 | 22.15 | 2-Oxysebacic acid |  |  | 0.197 | Dicarboxylic acids known to be antibacterial agents |
| 44 | 22.64 | 8,11-Octadecadienoic acid methyl ester |  |  | 0.317 | Antifungal properties (in all long chain fatty acids) |
| 45 | 22.79 | 4-methoxy-2-hydroxy -Cinnamic acid |  |  | 0.550 | Used in flavors and in some pharmaceuticals |
| 46 | 22.80 | 3-methoxy-4-(hydroxy)cinnamate |  | 0.848 |  | Used in flavors and in some pharmaceuticals |
| 47 | 23.21 | Inositol-O-acyllo |  |  | 0.265 | Sugar alcohol can be used to replace sugar but do not raise the blood sugar |
| 48 | 23.41 | N,O,O',O''-Uric acid |  |  | 0.378 |  |
| 49 | 23.71 | 3,4-bis(hydroxy)cinnamate |  | 1.114 | 0.612 | Used in flavors and in some pharmaceuticals |
| 50 | 23.90 | Benzoic acid-2-[amino]-3-[hydroxy]-methylester |  | 0.572 |  | Benzoic acid and its derivatives are antibacterial agents |
| 51 | 24.85 | 9,12 Octadecadienoic acid (Z,Z) |  | 0.173 | 0.205 | Linoleic acid is an essential fatty acid that must be consumed for proper health, antimicrobial, anti-inflammatory |
| 52 | 24.95 | 11-Cis-Octadecenoic acid |  | 0.318 | 0.134 | Antifungal properties (in all long chain fatty acids) |
| 53 | 25.43 | Octadecanoic acid |  | 0.603 |  | Antifungal properties (in all long chain fatty acids) |
| 54 | 26.65 | 4-Penten-1-one,1,5-diphenyl |  | 0.557 |  | Antifungal |
| 55 | 28.06 | Dehydroabietic acid |  | 0.528 |  |  |
| 56 | 28.48 | Phenol, 2, 2'-methylenebis[6-(1,1'-dimethylethyl)-4-methyl-] | 16.754 |  |  |  |
| 57 | 30.46 | 2-Hydroxyasebacic acid |  | 0.293 |  | Dicarboxylic acids known to be antibacterial agents |
| 58 | 30.56 | 1,2-Benzenedicaboxylic acid,diisocotyl ester | 16.222 | 0.235 |  | Dicarboxylic acids known to be antibacterial agents |
| 59 | 30.63 | [[(3α,5α,11β,20S)-pregnane-3,11,20,21tetraly]tetra(hydroxy) |  | 0.370 |  | A derivative of pregnane a female sex hormone. Hormone-like maybe precursor to female hormone, estrogen,can be used to improve female fertility |
| 60 | 31.38 | 2,3-bis(hydroxy )-Hexadecanoic acid-propyl ester |  | 0.456 | 0.140 | Antifungal properties (in all long chain fatty acids) |
| 61 | 31.99 | 2-Hydroxy sebacic acid |  | 0.149 | 0.181 | Dicarboxylic acids known to be antibacterial agents |
| 62 | 32.23 | 2-Hydroxy heptanoic acid |  | 0.711 |  | Antifungal properties (in all long chain fatty acids) |
| 63 | 33.41 | Androsta-3,5-diene-3,17-diol,17acetyl-3-O-(t-butyldimethyl)- |  | 0.370 |  | A derivative of the male hormone androgen can be a precursor to androgen |
| 64 | 34.32 | 2,3-bis[hydroxy]-Octadecanoic acid, propyl ester |  | 1.862 |  | Antifungal properties (in all long chain fatty acids) |
| 65 | 35.20 | Rhodaxanthin* |  | 0.084 |  |  |
| 66 | 35.77 | [(2-(3,4-bis[(hydroxyphenyl]-3,4-dihydro-2H-1-benzopyran-3,5,7-triyl) tris(quercetine) |  | 0.296 |  | Antioxidant |
| 67 | 20.46 | 3,4,5-tris(hydroxy )Benzoic acid-ester |  | 2.291 | 2.694 | Benzoic acid and its derivatives are antibacterial agents |
